# Reappearance of Symptoms after GPi‐DBS Discontinuation in Cervical Dystonia

**DOI:** 10.1002/mdc3.13162

**Published:** 2021-02-26

**Authors:** Emma A. Honkanen, Jaana Korpela, Eero Pekkonen, Valtteri Kaasinen, Martin M. Reich, Juho Joutsa

**Affiliations:** ^1^ Clinical Neurosciences University of Turku Turku Finland; ^2^ Division of Clinical Neurosciences Turku University Hospital Turku Finland; ^3^ Department of Neurology Satasairaala Central Hospital Pori Finland; ^4^ Turku PET Centre Turku University Hospital Turku Finland; ^5^ Department of Neurology, Helsinki University Hospital and Department of Clinical Neurosciences (Neurology) University of Helsinki Helsinki Finland; ^6^ Department of Neurology University Hospital and Julius Maximilian University Würzburg Germany; ^7^ Turku Brain and Mind Center University of Turku Turku Finland

**Keywords:** dystonia; deep brain stimulation; internal globus pallidus; neuromodulation; brain stimulation

## Abstract

**Background:**

Deep brain stimulation of the globus pallidus interna (GPi‐DBS) is a highly efficacious treatment for cervical dystonia. Typically, the treatment response is delayed, appearing and increasing even months after implantation. However, it is not known how fast the symptoms reappear and whether there is a long‐term therapeutic effect after the stimulation is discontinued.

**Objectives:**

To study symptom reappearance after switching GPi‐DBS off in cervical dystonia.

**Methods:**

Twelve patients with bilateral GPi‐DBS were included in the study. The Toronto Western Spasmodic Torticollis Rating Scale (TWSTRS) was evaluated during the study with DBS stimulation on, after switching the stimulation off and 2 days after the stimulation was switched off. Presurgical symptom severity and best postsurgical response were extracted from the hospital records.

**Results:**

At the time of the investigation, GPi‐DBS was associated with 67 (SD 39)% symptom improvement of presurgical symptoms severity (*P* = 0.001). Symptom improvement decreased to 27 (53)% (*P* = 0.046) (n = 12) acutely after switching the stimulation off and was further reduced to 4 (56)% 2 days after discontinuation (*P* = 0.01) (n = 11), reaching the presurgical level (*P* = 0.42). In descriptive analyses, older age was associated with faster worsening of symptoms (*P* < 0.05). Presurgical symptoms severity, stimulation parameters or magnitude of treatment response did not predict symptom worsening. All but one patient tolerated 2 days DBS switched off.

**Conclusions:**

The results provide novel information about the time frame and severity of symptom worsening after discontinuing GPi‐DBS in cervical dystonia. Symptoms partially reappear immediately after discontinuing GPi‐DBS and full presurgical symptom severity is reached within 2 days.

Deep brain stimulation (DBS) of the globus pallidus interna (GPi) is a highly efficacious treatment for cervical dystonia, resulting in approximately 65% symptom reduction.^1^, ^2^ However, the mechanisms of the therapeutic effects of GPi‐DBS in dystonia are not completely understood. In contrast to Parkinson's disease or essential tremor, the therapeutic effect of DBS in dystonia occurs slowly over time, reaching the maximum level typically months after device implantation.^3^, ^4^ These delayed effects have been attributed to neuroplastic changes caused by the stimulation.^5^ This effect differs from the immediate clinical effects of DBS for example in essential tremor, which are thought to result from direct disruption of the abnormal network activity.^6^, ^7^

Given that it may take several months until the optimal treatment response is achieved, it has been hypothesized that the clinical effects also disappear slowly after discontinuing the stimulation. In generalized dystonia, there may be sustained benefit after discontinuation of the stimulation but the evidence is mixed.^5^, ^8^, ^9^, ^10^, ^11^, ^12^ The symptoms in cervical dystonia have been reported to return very soon after the discontinuation of the stimulation and plateau with no additional worsening during the next 5 hours.^13^ However, given the limited presurgical information and short follow‐up period, it is not known whether these patients reached presurgical symptom severity or whether their symptoms would have further worsened in a longer off stimulation follow‐up. Evaluating the temporal course of symptom reappearance is clinically relevant to understand the effects of unintentional or intentional disruption of DBS treatment and for research studies investigating the neurobiological effects of DBS.

The aim of this study was to evaluate the worsening of cervical dystonia symptoms after switching GPi‐DBS off as measured at the two‐day follow‐up. Our hypothesis was that the immediate symptom reappearance is partial and that there is also a slower component of symptom worsening.

## Methods

### Patients

Twelve dystonia patients with bilateral GPi‐DBS treatment participated into the study. All patients had isolated cervical dystonia at the time of diagnosis. However, as focal dystonias frequently spread to other body parts,^14^ our two patients (patients 11 and 12) exhibited spread of dystonia symptoms to segmental dystonia (the affected body parts were neck, mouth and eyes) or generalized dystonia (the affected body parts were neck, mouth, vocal cords, trunk, left upper limb and left lower limb). Ten (83%) of 12 patients remained as isolated cervical dystonia at the time of the study. In all cases, treating clinicians had referred patients to DBS center due to of troubling cervical dystonia and insufficient response to other treatments like botulinum toxin injections. All patients were implanted with bilateral GPi‐DBS electrodes (Medtronic Activa PC, n = 8; Abbott St. Jude Infinity, n = 3; or Boston Scientific Vercise, n = 1). Voltages (V) in devices operating in current mode (Abbott) were calculated using current (mA) and resistance (ohms). This information was not available for the Boston Scientific device (n = 1).

### Study Design and Data Collection

The Mini‐Mental State Examination (MMSE) was performed with DBS stimulation on (ON) to exclude cognitive impairment. The effects of the discontinuation of DBS were studied by switching the stimulation off for 2 days. Clinical evaluation was performed during normal stimulation (ON), immediately (≈10–30 minutes) after the stimulation was turned off (OFFacute) and 2 days after DBS was switched off (OFFchronic). During the OFFacute visit, patients were monitored for 2 hours to record side effects and rapid changes in dystonia symptom severity. To test and control for assessment order, the order of ON and OFF visits was counterbalanced between the subjects (eight subjects were examined first under ON and four were examined under OFFacute conditions). OFFchronic was always performed after OFFacute to avoid switching the device on and off repeatedly. If OFF conditions were performed before the ON condition, the ON condition was performed at least 2 weeks after switching the stimulation back on. Side effects after DBS was switched back on were evaluated in a clinical interview and examination at the end of OFFchronic visit and followed up until resolved. All subjects confirmed reaching their original DBS clinical effect within 3 days of switching the stimulation on again.

Eleven patients completed the whole study. One patient developed abrupt worsening of the dystonia when DBS was switched off, and DBS was reinitiated within 10 minutes as requested by the patient. Therefore, the OFFchronic evaluations included 11 patients, and all other assessment points included 12 patients. The severity of dystonia was assessed using the Toronto Western Spasmodic Torticollis Rating Scale (TWSTRS)^15^ (severity scale range 0–35 points, disability scale 0–30 points, pain scale 0–20 points, total 0–85 points) and the Burke‐Fahn‐Marsden Dystonia Rating Scale (BFMDRS)^16^ movement part (range 0–120 points) during the ON, OFFacute and OFFchronic visits. All clinical assessments during the study were performed unblinded by the same investigator (E.A.H.). Clinical information for presurgical symptom severity, best postsurgical response, delay to best response and dominant preoperative symptom type (phasic/tonic as previously described^9^) were extracted from the hospital records.

### Statistical Analysis

Mean, standard deviation (SD) and range were calculated to describe the demographic data. TWSTRS and BFMDRS scores were compared between time points using paired samples t‐tests. All statistical testing was performed using TWSTRS scores, but percentage changes from the presurgical symptoms level are also presented to better illustrate the changes of dystonia severity. Any effects of assessment order (ON or OFF first) were excluded by comparing total and change of evaluation scores using Mann–Whitney *U* tests. Characteristics of the subgroups were compared using Mann–Whitney *U* tests and Fisher's exact test as appropriate. Additional descriptive analyses were conducted to identify demographic and clinical characteristics that could predict the severity of symptom reappearance after discontinuing DBS. These analyses were performed by calculating Spearman rank order correlation or a point‐biserial correlation coefficients. IBM SPSS Statistics (version 26, SPSS Inc., Chicago, IL, USA) was used for statistical analyses. *P*‐values less than 0.05 were considered significant.

## Results

The demographic data and DBS parameters at the group level are shown in Table 1. Six (50%) patients got botulinum toxins injections for the cervical dystonia at the time of DBS implantation and 3 (25%) patients at the time of the study. All patients (n = 12) used benzodiazepines before and after DBS implantation. All patients had monopolar stimulation and one active contact on both sides. Eleven patients had omnidirectional stimulation and one patient had directional stimulation.

**TABLE 1:** Demographic characteristics of patients and parameters of DBS

		All patients (n = 12)	Cervical only (n = 10)
Sex (female/male)		6/6	6/4
Age (mean (SD) [range])		52.8 (8.1) [41–68]	53.1 (8.6) [41–68]
Age of onset		40.7 (6.5) [27–49]	41.0 (6.3) [27–49]
Age at the surgery		50.1 (7.3) [37–62]	50.3 (8.0) [37–62]
Duration of DBS treatment (years)		2.8 (1.7) [0.33–5.25]	2.8 (1.7) [0.33–5.25]
GPi‐DBS	Amplitude (V) (mean (SD) [range])	2.7 (0.7) [1.8–3.7] (n = 11)	2.6 (0.8) [1.8–3.7] (n = 9)
	Pulse Width (us)	208 (85) [90–330]	220 (88) [90–330]
	Frequency (Hz)	83 (42) [40–130]	82 (41) [50–130]

**FIG. 1:**
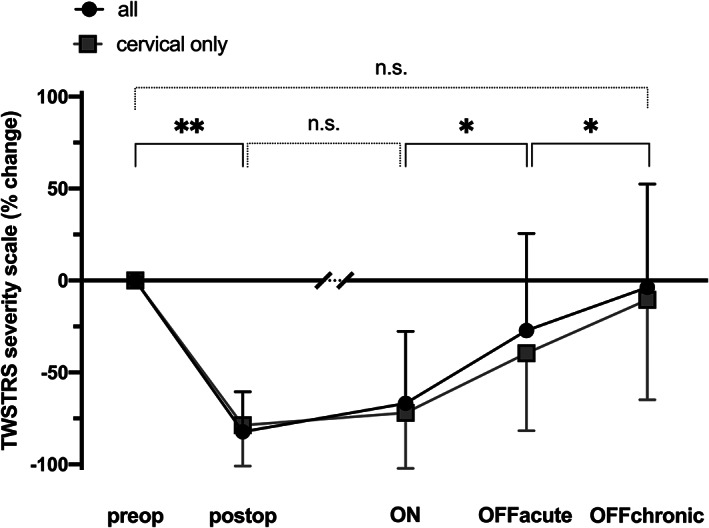
Mean and standard deviation of percentage changes of TWSTRS severity scale after the surgery and during the study with stimulation on (ON), acutely after switching stimulation off (OFFacute) and two days after discontinuation (OFFchronic). Differences between the absolute TWSTRS scores are marked with ** (*P* < 0.01), * (*P* < 0.05) or n.s. (non‐significant). TWSTRS = Toronto Western spasmodic torticollis rating scale.

None of the patients showed cognitive impairment and the mean MMSE score was 28.7 points (SD 0.9, range 27–30 points). On average, the clinical response to GPi‐DBS was an 82 (SD 22)% reduction on TWSTRS severity scale [mean (SD) TWSTRS severity scale preoperative 15.8 (SD 7.6) vs postoperative 3.6 (SD 4.7) points, *P* < 0.001] (Fig. 1) and the mean delay to maximal response was 5.6 (SD 4.4, range 1–12) months.

At the time of the investigation, the treatment response was maintained at 67 (SD 39)% improvement from baseline with no significant change from the symptom severity for best clinical response [mean (SD) TWSTRS severity scale ON 4.9 (SD 5.6) vs postop 3.6 (SD 4.7) points, *P* = 0.47] (Fig. 1). After stimulation was discontinued (OFFacute), clinical improvement from presurgical symptom severity was reduced to 27 (53)% [ON 4.9 (5.6) vs OFFacute 10.2 (7.7) points, *P* = 0.046] (Fig. 1). At 2 days follow‐up (OFFchronic), symptoms were further worsened to 4 (56)% [OFFacute 8.5 (5.6) vs. OFFchronic 13.7 (7.4) points, *P* = 0.01, n = 11] and no longer differed from presurgical symptom severity [OFFchronic 13.7 (7.4) vs preop 15.8 (8.0) points, *P* = 0.42] (Fig. 1). In patients with no spread of dystonia (n = 10), the results remained the same. The best treatment response [79 (22)%] was maintained at ON [72 (30)%]. At OFFacute, clinical improvement was reduced to 39 (42)% [ON 4.6 (5.4) vs OFFacute 8.5 (5.9) points, *P* = 0.004] and further reduced to 10 (54)% at OFFchronic [OFFacute 8.5 (5.9) vs OFFchronic 13.3 (7.6) points, *P* = 0.02], which did not differ significantly from presurgical symptom severity [OFFchronic 13.3 (7.6) vs preop 16.3 (8.3) points, *P* = 0.27] (Fig. 1).

**FIG. 2:**
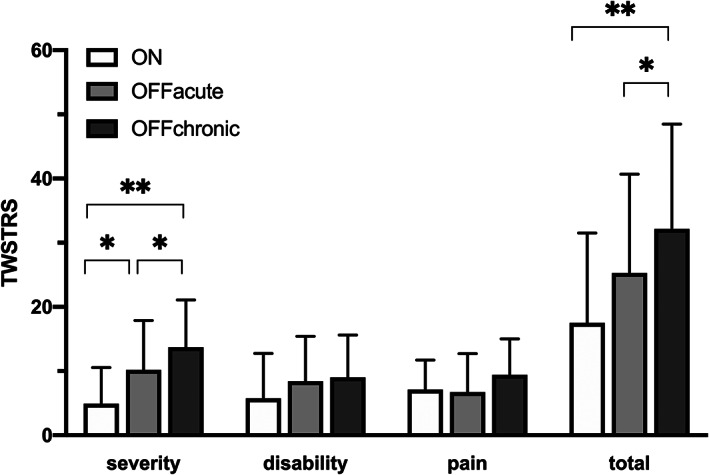
Mean and standard deviation of TWSTRS scores during the study with stimulation on (ON), acutely after switching stimulation off (OFFacute) and two days after discontinuation (OFFchronic). Differences between the scores are marked with ** (*P* < 0.01) or * (*P* < 0.05). TWSTRS = Toronto Western spasmodic torticollis rating scale.

In parallel with the TWSTRS severity scale, the TWSTRS total score increased after discontinuing the stimulation (Fig. 2, Table 2). However, no significant differences in TWSTRS disability or pain scales were noted between ON, OFFacute and OFFchronic conditions (Fig. 2, Table 2). Disability scores increased slightly when comparing the ON and the OFFchronic conditions but the change remained nonsignificant (*P* = 0.06) (Fig. 2, Table 2). In patients with no spread of dystonia symptoms (n = 10), the results remained essentially the same. Specifically, the TWSTRS total score increased from the ON to OFFchronic condition (*P* = 0.008) but the difference between the OFFacute and OFFchronic barely failed to reach significance (*P* = 0.06).

**TABLE 2:** Individual clinical scores of patients

Patient	TWSTRS preop	TWSTRS postop	TWSTRS ON				TWSTRS OFFacute				TWSTRS OFFchronic				BFMDRS ON	BFMDRS OFFacute	BFMDRS OFFchronic
	Severity Scale	Severity Scale	Severity Scale	Disability Scale	Pain Scale	Total	Severity Scale	Disability Scale	Pain Scale	Total	Severity Scale	Disability Scale	Pain Scale	Total			
1	11	0	1	0	0,5	1,5	1	1	5,75	7,75	18	7	7,25	32,25	0	0	3
2	34	11	14	23	8,25	45,25	16	18	11,25	45,25	25	22	13,5	60,5	4,5	4,5	8
3	17	2	0	0	0	0	1	0	1	2	2	0	1,25	3,25	0	0	0
4	23	13	0	2	9,75	11,75	10	11	3	24	18	14	10,5	42,5	0	2	6
5	21	2	0	2	9,25	11,25	2	0	0	2	6	4	8,25	18,25	0	0	1
6	4	0	0	0	9	9	4	11	17,5	32,5	2	4	17,25	23,25	0	0,5	0
7	15	3	10	6	8	24	13	4	12,25	29,25	13	4	8	25	3	0	3
8	15	9	11	4	10,75	25,75	14	4	7,75	25,75	15	4	0	19	4,5	7	7,5
9	13	3	4	11	11,5	26,5	13	12	9	34	16	13	14,5	43,5	0,5	3	4,5
10	10	0	6	12	5,25	23,25	11	9	0	20	18	12	7,25	37,25	0	0	6
11	15	0	0	0	0	0	28	22	0	50	‐	‐	‐	‐	0	18	‐
12	11	0	13	9	13	35	9	9	13	31	18	15	15,75	48,75	3	3	10
mean median (SD)	15.8 15.0 (7.6)	3.6 2.0 (4.7)	4.9 2.5 (5.6)	5.8 3.0 (7.0)	7.1 8.6 (4.6)	17.5 17.5 (14.0)	10.2 10.5 (7.7)	8.4 9.0 (7.0)	6.7 6.7 (6.0)	25.0 27.5 (15.1)	13.7 16.0 (7.4)	9.0 7.0 (6.6)	9.4 8.3 (5.6)	32.1 32.3 (16.3)	1.3 0 (1.9)	3.2 1.3 (5.2)	4.5 4.5 (3.4)

BFMDRS, Burke‐Fahn‐Marsden Dystonia Rating Scale; TWSTRS, Toronto Western Spasmodic Torticollis Rating Scale.

BFMDRS movement scores were significantly lower at the ON compared to OFFchronic visits (*P* = 0.003) and the OFFacute compared to OFFchronic visits (*P* = 0.005) (Table 2). In patients with no spread of dystonia symptoms (n = 10), similar differences were found between the ON and OFFchronic visits (*P* = 0.006) and between the OFFacute and OFFchronic visits (*P* = 0.008).

The order of ON/OFF assessments did not have a significant effect on any of the TWSTRS or BFMDRS raw scores (all *P*‐values>0.2). In addition, relative or total changes in TWSTRS scores between evaluations were not affected by the order (all *P*‐values>0.1).

In the descriptive analyses, patients with milder acute symptom worsening had younger age of dystonia onset (r = 0.71, n = 12, *P* = 0.01), and were younger at the time of surgery (r = 0.60, *P* = 0.04) and at the time of the study (r = 0.63, *P* = 0.03). However, no significant correlations were noted between the TWSTRS changes and presurgical dystonia symptom severity, symptom type (phasic vs tonic), DBS parameters, or treatment duration or response.

After DBS was turned back on to the original settings, four (33%) patients exhibited short‐term side effects immediately after restarting the stimulation. The side effects included nausea, paresthesia, speech problems and dizziness, which were clearly reduced in less than 10 minutes and resolved completely within 30 minutes. Patient 11 with rapid clinical worsening, who did not tolerate the stimulation OFF condition, did not show any side effects when the stimulation was switched back on. In the four patients with side effects, DBS amplitude was higher compared with the amplitude of the patients without side‐effects (median 3.5 V (range 2.3–3.7 V) (n = 4) vs 2.1 V (1.8–3.2 V) V (n = 7), *P* = 0.04). However, there were no differences in age, DBS interval, TWSTRS or BFMDRS scores or other DBS parameters between patients with and without side‐effects (all *P*‐values>0.1). To assess the delay in therapeutic effect reappearance, the patients were asked about the relief of dystonic symptoms after DBS was switched back on. Four (33%) of patients reported reaching their complete response in less than 30 minutes, and remainder of the patients reported to have reached their complete response within 3 days.

## Discussion

The main finding of this study is that symptoms of cervical dystonia reappear in two phases after discontinuation of GPi‐DBS. Symptoms partially reappear immediately after discontinuing the stimulation and further worsen, reaching the preoperative level within 2 days after discontinuing the stimulation. Symptom reappearance is slower in younger patients but is not dependent on the stimulation settings. However, stimulation‐related side effects were dependent on the stimulation settings and were more common in patients with higher stimulation amplitude. Almost all patients tolerated the discontinuation of DBS well. These results provide novel clinical information about the tolerability and symptom reappearance after discontinuing GPi‐DBS treatment in cervical dystonia.

In the only previous study on this topic in cervical dystonia, Levin et al reported a carefully controlled study investigating timeframe of symptom worsening after discontinuation of GPi‐DBS.^13^ In their study, the symptoms reappeared within 10 minutes after discontinuation of the stimulation and were not further worsened during the 5‐hour follow‐up with the stimulation off. Our results are consistent with these findings showing immediate worsening of the symptoms with no changes during the following 2 hours. However, in our study, the symptoms further worsened and reached the level of presurgical symptom severity later, as measured 2 days after discontinuation of the stimulation, which was not investigated in the previous study. These findings indicate that there are two distinct components, namely, fast and slow, in symptom reappearance after discontinuing stimulation.

Patients who were younger at dystonia onset, DBS operation, and time of this study showed slower symptom reappearance than older patients. As these measures are highly correlated, it is not possible to identify which of these variables is driving this effect. Younger age has previously been shown to be predictor for sustained DBS effect reported in primary generalized dystonia.^17^ Specifically, patients with higher preoperative plasticity (as measured with paired associative stimulation using transcranial magnetic stimulation) have a better treatment response,^18^ and the treatment response in patients with high plasticity is prolonged after cessation of DBS treatment.^5^ Similarly, brain lesions may cause cervical dystonia with delays of months or even years, indicating that slow neuroplastic changes are involved in the neurobiological mechanisms of dystonia.^19^ Thus, slow changes in clinical symptoms are likely to be mediated via neuroplastic changes rather than direct disruption/excitation/inhibition of neural signaling by DBS.

Differences between cervical (focal), segmental and generalized dystonia may be linked to different pathophysiological mechanism, which can also be reflected in clinical outcomes of DBS treatment.^20^ These mechanisms may also be reflected in symptom reappearance after discontinuing DBS across different clinical phenotypes of dystonia. In segmental dystonia, discontinuation of GPi‐DBS lead to rapid symptom return, reaching the preoperative level already in 4 hours.^9^ Interestingly, this finding was also observed in our only segmental dystonia patient who showed rapid symptom worsening above the preoperative level immediately after switching DBS off. Symptom worsening above the presurgical level can result from disease progression or a rebound effect, which may have resolved with follow up.^21^ In generalized dystonia covering heterogeneous etiologies, the outcomes after discontinuing DBS are highly variable ranging from immediate symptom return up to sustained benefit.^5^, ^8^, ^10^, ^11^, ^12^

In our study, a clear clinical worsening after DBS was switched off was observed in the TWSTRS severity scale but not in the other measures. Pain and disability ratings were higher at the two‐day follow‐up but these effects did not reach a level of significance. The TWSTRS disability scale measures impairment in daily activity and therefore is likely not sensitive to short‐term changes, as in the present study. Pain in dystonia has been considered to be correlated with motor symptom severity,^22^ but there were no significant differences in the pain ratings between ON and OFF conditions in the present study. Thus, it is possible that pain increases slower after the motor symptoms have become worse. However, similar to the disability scale, the TWSTR pain scale may not be sensitive to short‐term changes, and these findings should be interpreted with caution.

When reinitiating DBS stimulation, 33% of our patients reported transient side effects, including nausea, speech problems, paresthesia and dizziness. Discontinuation of DBS for 2 days was tolerated by all but one patient, in whom symptoms spread as in segmental dystonia. Side effects were associated with higher stimulation amplitudes but not with any demographic or clinical data. Dysarthria, which is the most common GPi stimulation‐induced adverse effect,^23^ was also the most commonly reported side effect after DBS was switched back on in the present study. These data support the feasibility of study designs or clinical interventions that require switching DBS off and on for up to 2 days. However, careful monitoring for rapid symptom worsening and warning patients about transient side effects after reinitiating the stimulation are recommended.

There are some limitations in our study that should be considered when interpreting the results. First, although the sample size in our study was comparable to previous studies in the field,^9^, ^10^, ^11^, ^13^ we may not have had sufficient statistical power to detect subtle changes in pain or disability severity or weak associations between symptom reappearance and clinical parameters. Second, the study design did not allow for blinding the patients or investigators regarding the order of the visits. However, the order of the ON and OFF visits was pseudorandomized. Third, although all ON and OFF clinical evaluations were performed by a single clinical investigator (E.A.H.), preoperative and best postoperative TWSTRS severity scale scores were extracted from the hospital records retrospectively, which may reflect interrater differences between the scores. However, postoperative scores did not differ from ON scores, suggesting that there was no major systematic difference between the raters. DBS treatment time also varied between the patients, but all had reached a clear and stable response after the implantation. Fourth, other symptoms of dystonia that are not included in the TWSTRS symptom severity score, such as tremor or myoclonus, were not investigated in the present study. These symptoms could be mediated via separate brain networks and worsened independently of overall dystonia symptom severity measured with TWSTRS. Finally, in this study, the dystonia symptoms reached the preoperative level after 2 days of DBS discontinuation, but it is not known whether the symptoms would have further worsened, beyond the presurgical levels, with longer follow‐up.

To conclude, this is the first study to investigate symptom worsening for more than 5 hours after temporarily discontinuing DBS treatment in cervical dystonia. Combined with prior work,^13^ our results demonstrate a two‐phase reappearance of the clinical symptoms, which on average reached presurgical symptom severity within 2 days. Rapid symptom worsening was associated with older age, whereas other variables did not seem to predict the worsening. Discontinuing the stimulation was well tolerated by >90% of all patients (100% of the patients with cervical dystonia only), and switching stimulation back on caused only relatively minor, transient side effects, supporting the feasibility of study designs that require discontinuation of DBS in cervical dystonia.

## Author Roles

(1) Research project: A. Conception, B. Organization, C. Execution; (2) Statistical analysis: A. Design, B. Execution, C. Review and critique; (3) Manuscript preparation: A. Writing the first draft, B. Review and critique.

E.A.H: 1B, 1C, 2B, 3A.

J.K: 1B, 3B.

E.P: 1B, 3B,

V.K: 1B, 3B.

M.M.R: 1B, 3B.

J.J: 1A, 1B, 1C, 2A, 2C, 3B.

## Disclosures

### Ethical Compliance Statement

The study was approved by the Ethics Committee of Hospital District of Southwest Finland (ETMK Dnro: 135 /1801/2018). The study was conducted according to the Declaration of Helsinki. Written informed consent was obtained from all participants. We confirm that we have read the Journal's position on issues involved in ethical publication and affirm that this work is consistent with those guidelines.

### Funding Sources and Conflict of Interest

The preparation of this article was financially supported by the Finnish Medical Foundation and Turku University Hospital (Finnish governmental research funding, VTR‐funds). The authors declare that there are no conflicts of interest relevant to this work.

### Financial Disclosures for the previous 12 months

Dr. Honkanen has received funding from the Turku University Foundation, Turku University Hospital (Finnish governmental research funding, VTR‐funds) and The Finnish Parkinson Foundation. Dr. Korpela declares that there are no additional disclosures to report. Dr. Pekkonen reports following disclosures: Consulting neurologist for Finnish Patient Insurance Centre. Standing Member of the MDS Non‐Motor Parkinson's Disease Study Group. Person Responsible of Trial in Finland: International DYSCOVER‐study (Dyskinesia Comparative Interventional Trial on Duodopa vs. oral medication) 2017–19, Organized by Abbvie, Consulting fees: NordicInfu Care AB, Abbvie, Zambon, Member of Advisory board: Abbvie, Lecture fees: Abbott, Abbvie, Medtronic, Orion. Travel support: Abbott, Abbvie, Boston Scientific. Dr. Kaasinen reports following disclosures: Honoraria for lecturing (Abbvie, Nordic Infucare), Advisory Board (Abbvie, Nordic Infucare), Travel Expenses (Nordic Infucare). Dr. Reich has received grant support and honoraria for speaking from Medtronic and Boston Scientific, outside the submitted work. Dr. Reich's position is funded by the Deutsche Forschungsgemeinschaft (DFG, German Research Foundation) – Project‐ID 424778381 – TRR 295. Dr. Joutsa has received grants/awards from the Instrumentarium Research Foundation, Finnish Foundation for Alcohol Studies and the Medical Society Duodecim.
